# Multimodal imaging in a patient with combined hamartoma of the retina and retina pigment epithelium

**DOI:** 10.5935/0004-2749.20220027

**Published:** 2022

**Authors:** José Mauricio Botto Garcia, Hugo Mendes Silva, David Leonardo Cruvinel Isaac, Marcos Pereira Ávila

**Affiliations:** 1 Reference Center in Ophthlamology, Universidade Federal de Goiás, Goiânia, Brazil

**Keywords:** Hamartoma/diagnosis, Retinal pigment epithelium, Retinal neoplasm, Tomography, optical coherence, Angiography, Humans, Case report, Hamartoma/diagnóstico, Epitélio pigmentado da retina, Neoplasia da retina, Tomografia de coerência óptica, Angiografia, Humanos, Relato de caso

## Abstract

Combined hamartoma of the retina and retinal pigment epithelium is a rare, benign intraocular tumor. Hamartoma of the retina and retinal pigment epithelium has been described in the literature as a condition presenting with variable retinal damage, ranging from partial epiretinal involvement to complete distortion of the retinal layers and retinal pigment epithelium. We report the case of an 8-year-old girl presenting with longstanding strabismus who was diagnosed with Hamartoma of the retina and retinal pigment epithelium based on multimodal imaging assessment. We explored the particular imaging findings from studies using spectral-domain optical coherence tomography, fundus autofluorescence, optical coherence tomography angiography, and fluorescein angiography.

## Abstract (pt)

O hamartoma combinado de retina e epitélio pigmentar da retina consiste em um tumor intraocular raro com comportamento benigno. O hamartoma combinado de retina e epitélio pigmentar da retina foi descrito na literatura apresentando dano retiniano variável, desde o envolvimento epirretiniano parcial até distorção completa das camadas retinianas e do epitélio pigmentar da retina. Relatamos o caso de uma menina de 8 anos com estrabismo de longa data que foi diagnosticada com hamartoma combinado de retina e epitélio pigmentar da retina, com base na avaliação de imagem multimodal. Exploramos os achados de imagem específicos de estudos usando tomografia de coerência óptica de domínio espectral, autofluorescência, angiografia por tomografia de coerência óptica e angiografia fluorescente.

## INTRODUCTION

Combined hamartoma of the retina and retinal pigment epithelium (CHRRPE) is a rare, benign, presumably congenital, intraocular tumor with classic clinical features^(1)^. In 1984, Schachat et al.^(2)^ described it as a lesion with major pigmentation, elevation, vascular tortuosity, and vitreoretinal interface changes.

Gass et al.^(1-3)^ divided CHRRPE into two histological sub-categories as per optic nerve involvement. The one that does not affect the optic nerve head shows lack of retinal pigmented epithelium (RPE) migration, less prominent RPE hypertrophy or retinal capillary proliferation, and disorganization in the layer’s retina. Further models it is based on the extent of retinal damage^(4)^.

Studies based on spectral domain optical coherence tomography (SD-OCT) reported focal or folded traction and inner retinal thickening without significant attenuation of the outer retina or RPE. Shields et al.,^(5)^ indicated that tumor involvement appeared to be limited to the inner retina, likely because deeper structures could not be clearly imaged. Nonetheless, multiple small hyperreflective triangular spots detected on structural SD-OCT located in the outer nuclear layer (ONL) (“shark-teeth” sign) might indicate a certain degree of outer retina compromise along the edges of CHRRPE, without a back-shadowing phenomenon^(6)^.

Optical coherence tomography angiography (OCT angiography) imaging enables the outlining of the CHRRPE structure^(6-8)^. Qualitative and quantitative analyses have shown global disorganization of the superficial capillary plexus (SCP), deep capillary plexus (DCP), and choriocapillaris without inherent intraretinal microcirculation patterns of CHRRPEs^(2,5,6)^.

We report the case of a young patient diagnosed with CHRRPE using multimodal imaging based on SD-OCT, fundus autofluorescence, fluorescein angiography, and OCT angiography. We particularly explored the findings related to the inner and outer retinal changes with assessment using these technologies.

## CASE REPORT

**Figure 1:**
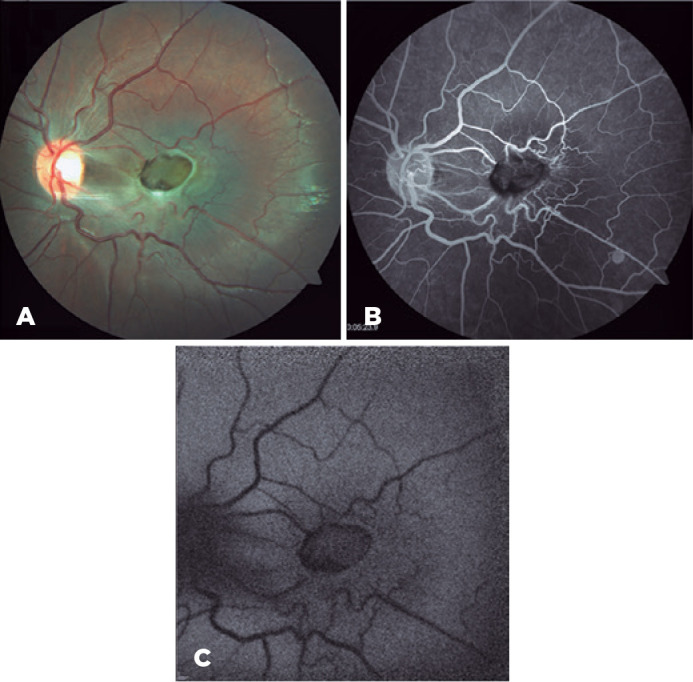
(A) Fundus photo demonstrating an oval, slightly elevated mass with hyperpigmentation and gliosis at the macula. The lesion spares the optic disc. (B) Mid-phase fluorescein angiography reveals hypofluorescence corresponding at the lesion site and perilesional vascular tortuosity with telangiectasia. (C) Fundus autofluorescence shows central hypoautofluorescence and obscured retinal microcirculation at the CHRRPE lesion.

An 8-year-old girl who had a history of persistent low vision and eye deviation in the left eye (OS) since the age of 6 y was referred to investigate an atypical fundus lesion identified by her pediatric ophthalmologist. Her medical history was unremarkable. On ocular examination, the best-corrected visual acuity was 20/25 in the right eye (OD) and 20/200 in the OS. Pupillary reflex, intraocular pressures, and anterior segment examination were within normal limits. The fundus examination revealed a rounded and slightly elevated hyperpigmented lesion at the macula, with gliosis. The surroundings of the lesion presented telangiectatic vessels without optic nerve involvement (Figure 1A).

Fluorescein angiography of the OS demonstrated early hypofluorescence adjacent to the hyperpigmentation and inner retinal tractional. There was marked tortuosity without late leakage (Figure 1B). Fundus autofluorescence displayed central hypoautofluorescence at the lesion site (Figure 1C).

**Figure 2:**
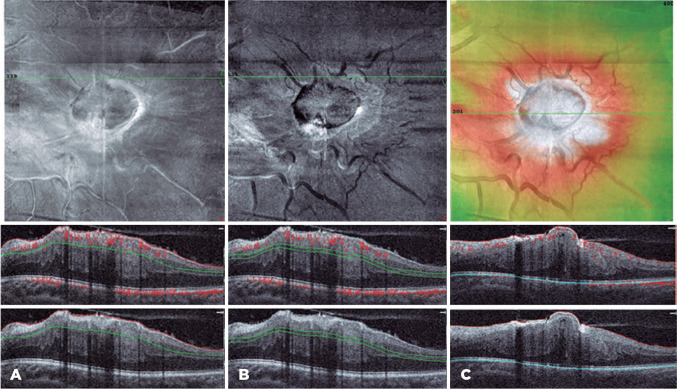
(A and B) SD-OCT B-scans en face structural with and without flow in SCP (a), DCP (b). En face flow map depicts rarefaction of all retinal plexuses. (B) En face structural overlapped by central thickness map showing central mass surrounded by increased retinal thickness. (C) Correspondent structural SD-OCT B-scan (total retina slab) with flow.

OCT angiography (RTVue XR Avanti; Optovue, Fremont, CA, USA), 3 x 3-mm volume scans with automated segmentation demonstrated rarefaction of all the retinal plexuses, with reduction in the superficial capillary plexus vessel density. It is important to highlight that this alteration may present as a projection artifact from the epiretinal membrane (ERM) and full-thickness retinal disorganization. *En face* structural imaging depicted increased tortuosity with global disorganization of the inner retina down to the choriocapillaris (Figure 2). The RPE was apparently intact around the edges of the lesion despite overlying hyperreflectivity caused by the tumor and distortion by an adjacent ERM.

The patient was diagnosed with CHRRPE, and ERM surgical procedure was indicated; however, the patient’s family refused treatment because of poor prognostic visual acuity and risk benefits. She was closely followed up with regular retinal imaging.

## DISCUSSION

CHRRPEs were first described as pigmented hamartomatous malformations of the retina, RPE, and overlying vitreoretinal interface. Combined hamartoma is usually diagnosed in young children, commonly with symptoms of strabismus or reduced visual acuity^(9)^. As shown in this case, one of the major differential diagnoses is classic ERM^(1)^. The distinction between CHRRPE and ERM relies mainly on the clinical history that reveals the absence of previous ocular inflammation, supported by the younger age of onset and specific OCT features^(8,10)^.

A frequent complication of CHRRPE includes retinal traction that may arise in about 80% of the patients, causing poor visual acuity^(1,10)^. Surgical repair may be effective in reducing the retinal damage and restoring vision, particularly in patients with a combination of features early diagnosed in young patients.

Enhanced depth imaging OCT has helped in differentiating the ERM-related CHRRPE in patients with focal traction in the form of a sawtooth (mini-peak) limited to the inner retina, from folded (maxi-peak) pattern, which promotes inward traction and deep retinal distortion^(1,10)^. Structural OCT allows the identification of omega-shaped disorganization of the inner retinal layers bounded posteriorly by the outer plexiform layer (omega sign), distinguishing CHRRPE lesions from idiopathic ERMs^(10)^. Chawla et al.,^(1)^ hypothesized that the extent of macular lesions is mostly limited by the OPL. Gupta et al.,^(8)^ described hyperreflective dots in the ONL on structural OCT B-scans of macular CHRRPEs that were referred to as the “shark-teeth” sign. Ellipsoid zone and RPE disruption appear to be common in peripapillary combined hamartomas^(1,6)^.

OCT angiography provides information on retinal vascular plexus without the risks of intravenous fluorescein dye^(6)^. In patients who are diagnosed with CHRRPE, OCT angiography reveals vascular network changes at the level of both, superficial and deep capillary plexus in the tumoral lesion^(6-8)^. Flow signals in the DCP constituting a filigree pattern are usually found in the peripapillary lesions, with full-thickness retinal disorganization and minimal preretinal fibrosis. However, a low density of the filigree pattern has been observed in macular lesions owing to partial thickness retinal involvement and disorganization by the dysplastic tissues^(8)^. This distinctive pattern helps in differentiating macular lesions from peripapillary lesions. Preretinal fibrosis (glial component) is present in most cases that reduces the vascular component.

CHRRPE lesions demonstrate partial, epiretinal involvement in a small group of patients; however, in others, it is remarkable for complete involvement of retinal and RPE involvement. Structural OCT is a fast-advancing imaging strategy that offers the possibility of analyzing the inner retinal layers and to some extent, the outer retina. OCT angiography imaging may be used regularly at some point, providing intraretinal microcirculation analysis for this tumor. The lack of long-term follow-ups precludes the analysis of changes over a long period of time in these lesions.
